# Supplementary figures and images for: Oncologist phenotypes and associations with response to a machine learning-based intervention to increase advance care planning: Secondary analysis of a randomized clinical trial
Source: PLoS One. 2022 May 27;17(5):e0267012. doi: 10.1371/journal.pone.0267012 (PMC9140236; doi:10.1371/journal.pone.0267012)

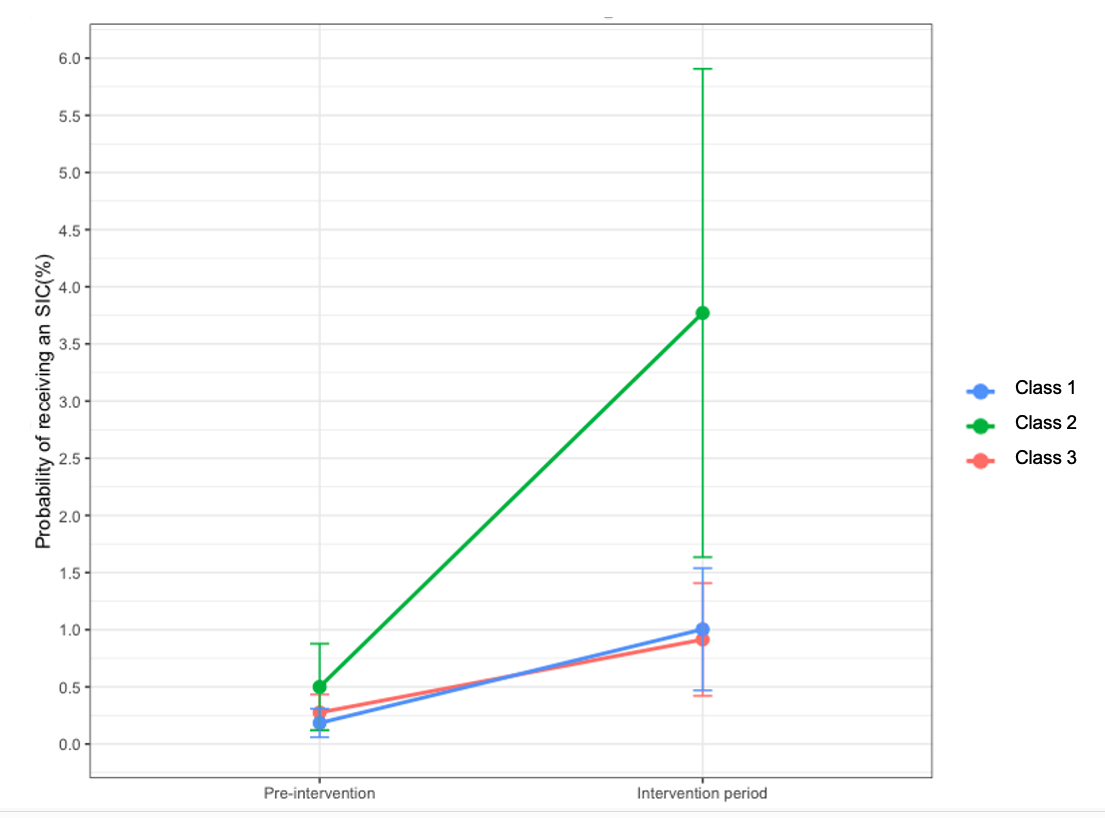

Supplement: S1 Fig — The adjusted probability of any patient in the cohort receiving an SIC during the pre-intervention and intervention periods by oncologist phenotype. Class 2 oncologists (green) had the highest response to the intervention, with the probability of receiving an SIC increasing from 0.5% during the pre-intervention period to 3.8% during the intervention period. The adjusted probability of ACP increased from 0.2% to 1.0% among class 1 oncologists, and from 0.3% to 0.9% for class 3 oncologists. (DOCX) [file pone.0267012.s002.docx]
