# Supplementary material for: Oncologist phenotypes and associations with response to a machine learning-based intervention to increase advance care planning: Secondary analysis of a randomized clinical trial
Source: PLoS One. 2022 May 27;17(5):e0267012. doi: 10.1371/journal.pone.0267012 (PMC9140236; doi:10.1371/journal.pone.0267012)
Supplement: S1 Table — (DOCX) [file pone.0267012.s003.docx]

**S1 Table: Model fit statistics by number of classes included in the model.**

| Number of latent classes | AIC | BIC | Entropy | Bootstrapped Likelihood Ratio Test (BLRT) |
| --- | --- | --- | --- | --- |
| 1 | 2748.25 | 2786.48 | 1.00 | --- |
| 2 | 2689.46 | 2748.54 | 0.97 | 0.010 |
| 3 | 2678.46 | 2758.40 | 0.96 | 0.010 |

Akaike information criteria (AIC), Bayesian information criteria (BIC), Entropy, and Bootstrapped likelihood ratio test (BLRT) to compare model fits by the number of classes included in the model. The model with 3 classes had the lowest AIC and highest clinical interpretability.
