# Supplementary material for: Oncologist phenotypes and associations with response to a machine learning-based intervention to increase advance care planning: Secondary analysis of a randomized clinical trial
Source: PLoS One. 2022 May 27;17(5):e0267012. doi: 10.1371/journal.pone.0267012 (PMC9140236; doi:10.1371/journal.pone.0267012)
Supplement: S2 Table — (DOCX) [file pone.0267012.s004.docx]

**S2 Table: Association between oncologist phenotype and response to nudges (whole cohort)**

| Phenotype | Oncologists, n (%) | Patients, n (%) | Adjusted probability of ACP, pre-intervention | Adjusted probability of ACP, intervention period | Percentage point difference in differences vs Class 3 (95% CI) | p-value |
| --- | --- | --- | --- | --- | --- | --- |
| Class 3 | 28 (67%) | 8656 (59.3%) | 0.3% | 0.9% | --- | --- |
| Class 1 | 9 (21%) | 5467 (37.4%) | 0.2% | 1.0%­­ | 0.2 (0,0.4) | 0.109 |
| Class 2 | 5 (12%) | 484 (3.3%) | 0.5% | 3.8% | 2.6 (0.9, 4.3) | 0.002 |
