# Supplementary material for: Oncologist phenotypes and associations with response to a machine learning-based intervention to increase advance care planning: Secondary analysis of a randomized clinical trial
Source: PLoS One. 2022 May 27;17(5):e0267012. doi: 10.1371/journal.pone.0267012 (PMC9140236; doi:10.1371/journal.pone.0267012)
Supplement: S3 Table — (DOCX) [file pone.0267012.s005.docx]

**S3 Table: Logistic regression at the patient-wedge level identifying clinician characteristics associated with increased likelihood of conducting an SIC:**

|  | OR | (95% CI) | | P |
| --- | --- | --- | --- | --- |
| **Sex** |  |  |  |  |
| *Female (ref)* | --- | --- | --- | --- |
| *Male* | 0.94 | 0.80 | 1.11 | 0.48 |
| **Age** |  |  |  |  |
| *18-35 (ref)* | --- | --- | --- | --- |
| *36-50* | 2.11 | 1.23 | 3.87 | 0.01 |
| *51-64* | 2.33 | 1.40 | 4.18 | 0.00 |
| *65+* | 2.18 | 1.27 | 4.00 | 0.01 |
| **Race** |  |  |  |  |
| *Black* | --- | --- | --- | --- |
| *White* | 1.05 | 0.85 | 1.29 | 0.67 |
| *Other* | 1.35 | 0.77 | 1.40 | 0.77 |
| **Insurance** |  |  |  |  |
| *Commercial* | --- | --- | --- | --- |
| *Medicaid* | 1.17 | 0.84 | 1.60 | 0.34 |
| *Medicare* | 1.29 | 1.03 | 1.63 | 0.03 |
| **Marital status** |  |  |  |  |
| *Married* | --- | --- | --- | --- |
| *Single* | 1.29 | 1.04 | 1.59 | 0.02 |
| *Other* | 1.35 | 1.11 | 1.64 | 0.00 |
| **CCI** |  |  |  |  |
| *0.00* | --- | --- | --- | --- |
| *1.00* | 1.99 | 0.55 | 7.20 | 0.28 |
| *>1* | 7.21 | 3.28 | 20.37 | 0.00 |
| **Oncology team** |  |  |  |  |
| *General (ref)* | --- | --- | --- | --- |
| *Breast* | 0.65 | 0.38 | 1.10 | 0.11 |
| *GI* | 3.40 | 2.29 | 5.04 | 0.00 |
| *GU* | 0.95 | 0.50 | 1.77 | 0.88 |
| *Lymphoma* | 1.81 | 1.10 | 2.92 | 0.02 |
| *Melanoma* | 3.73 | 1.93 | 7.20 | 0.00 |
| *Myeloma* | 2.11 | 1.13 | 3.92 | 0.02 |
| *Neuro* | 1.70 | 0.90 | 3.15 | 0.10 |
| *Thoracic* | 2.92 | 1.83 | 4.66 | 0.00 |
| **Clinician number of days in clinic per week** | 1.35 | 1.12 | 1.62 | 0.00 |
| **Clinician percentage of new patients per week** | 34.23 | 5.19 | 227.55 | 0.00 |
| **Clinician average patients per week** | 1.01 | 0.99 | 1.03 | 0.57 |
| **Clinician average encounters per day** | 0.98 | 0.91 | 1.06 | 0.68 |
| **Clinician years in practice** | 0.98 | 0.97 | 0.99 | 0.00 |
| **Clinician hospice enrollment rate** | 1.02 | 1.01 | 1.03 | 0.00 |
| **Clinician inpatient death rate** | 1.01 | 0.99 | 1.03 | 0.22 |
| **Clinician chemo rate** | 1.00 | 0.99 | 1.02 | 0.67 |
