# Supplementary material for: Oncologist phenotypes and associations with response to a machine learning-based intervention to increase advance care planning: Secondary analysis of a randomized clinical trial
Source: PLoS One. 2022 May 27;17(5):e0267012. doi: 10.1371/journal.pone.0267012 (PMC9140236; doi:10.1371/journal.pone.0267012)
Supplement: Attachment — Submitted filename: ResponseToReviewers_02222022.docx [file pone.0267012.s009.docx]

423 Guardian Drive	**Ravi B. Parikh, M.D., M.P.P.**

Blockley 1102	*Asst. Professor, Medical Ethics and Health Policy*

Philadelphia, Pennsylvania 19104	*Staff Physician, Phila. VA Medical Center*

Tel: 352.422.4285	Perelman School of Medicine

University of Pennsylvania

February 22, 2022

Randall J. Kimple MD, PhD

Academic Editor

PLOS ONE

Dear Dr. Kimple and Reviewers,

Thank you for your thoughtful review of our manuscript, “Oncologist Phenotypes and Associations with Response to a Machine Learning-Based Intervention to Increase Advance Care Planning: Secondary Analysis of a Randomized Clinical Trial.” We appreciate your comments and have responded to each of your concerns below. Manuscript revisions are highlighted in ***bold*** with page numbers indicating pages in the clean, revised version of the manuscript.

**REVIEWER 1 SPECIFIC COMMENTS**

1. **Although I understand why the authors labelled the physician clusters (as they wanted to make the clusters easier to interpret), I believe by labelling the clusters in the way that they did they actually made their results and analysis more misleading which in my opinion is a graver sin then making the results more difficult to interpret clinically. I would highly recommend that the authors change the labels of the clusters to cluster 1, cluster 2, and cluster 3 and simply explain what the doctors in the clusters tended to have in common rather than labelling them with demographics that the physicians tended to have in common. I think this would be a critical step in making sure that casual readers will better understand the methods and not misinterpret the results.**

We agree with the review on this important distinction. We have now amended the language throughout our manuscript to reflect this feedback. As suggested by the reviewer, we have now changed the labels of the clusters to cluster 1, cluster 2, and cluster 3, and describe the features shared by clinicians in each cluster, rather than directly labelling a cluster with the descriptive labels as before. Selected changes are shown below.

Abstract lines 41-52: *Three oncologist phenotypes were identified:* ***Class 1 (n=9) composed primarily of high-volume generalist oncologists, Class 2 (n=5) comprised primarily of low-volume specialist oncologists; and 3) Class 3 (n=28), composed primarily of high-volume specialist oncologists. Compared with class 1 and class 3, class 2*** *had lower mean clinic days per week (1.6 vs 2.5 [class 3] vs 4.4 [class 1]) a higher percentage of new patients per week (35% vs 21% vs 18%), higher baseline ACP rates (3.9% vs 1.6% vs 0.8%), and lower baseline rates of chemotherapy within 14 days of death (1.4% vs 6.5% vs 7.1%). Overall, ACP rates were 3.6% in the pre-intervention wedges and 15.2% in intervention wedges (11.6 percentage-­­­­point difference).* ***Compared to class 3, oncologists in class 1*** *(adjusted percentage-point difference-in-difference 3.6, 95% CI 1.0 to 6.1, p=0.006)* ***and class 2*** *(adjusted percentage-point difference-in-difference 12.3, 95% confidence interval [CI] 4.3 to 20.3, p=0.003) had greater response to the intervention.*

Results lines 316-322: *The probability of a high-risk patient (predicted 180-day mortality >10%) receiving an ACP increased significantly following the intervention among patients receiving care from* ***class 1 and class 2*** *oncologists compared to* ***class 3 oncologists****. Among patients receiving care from* ***class 3 oncologists,*** *the adjusted probability of a high-risk patient receiving an ACP increased from 2.3% pre-intervention to 7.6% during the intervention period. Among patients receiving care from* ***class 2 oncologists****, the adjusted probability of ACP increased from 3.1% pre-intervention to 20.7% in the intervention period (adjusted percentage-point difference-in-differences relative to* ***class 3 oncologists*** *12.3, 95% CI 4.3 to 20.3, p=0.003)* ***(Table 2)****.*

Discussion lines 438-441: *In particular, the intervention was associated with a 5.6-fold and 6.7-fold increase in response rates among* ***class 1 oncologists, who consisted primarily of general oncologists with higher patient volumes; and class 2 oncologists, who consisted primarily of specialists with lower patient volumes; compared to class 3 oncologists, who consisted primarily of specialists with higher patient volumes.***

**REVIEWER 2 SPECIFIC COMMENTS**

1. **I completely agree with the authors that the LPA can capture complex interactions across variables to generate classifications, but for this same reason, the conclusions and naming of the clusters in the manuscript is a bit discordant and oversimplify these same complex interactions. Drawing conclusions around volume and specialization without a more interpretable approach as supplementation (as emphasized in the abstract) risks the reader drawing incorrect conclusions about the data. For example, as stated in comment #5, the high-volume generalist cluster does not clearly show that high-volume generalists have a greater response, but rather there is a phenotypic group (which still has a substantial 33% representation by specialists) has a greater response to the intervention.**

Please see our response to Reviewer 1, point 1.

**I would really suggest to the authors to include simple regression models to help support their conclusions around these characteristics - this is also a common practice in computational health to provide simpler comparator models. Alternatively, I'd suggest the conclusions should otherwise be framed around the heterogeneous classes rather than the simpler specific volume/time availability characteristics.**

We thank the reviewer for this thoughtful suggestion. We’ve now included a logistic regression model as a supplemental analysis. We ran this regression model at the level of the patient wedge and measured the association between various clinician practice pattern characteristics and the likelihood of their patients receiving an SIC while adjusting for various patient level characteristics. Consistent with the results of our LPA analysis, we found that in general, patients seeing specialist oncologists were more likely to receive SICs, as were patients seeing clinicians with a higher number of clinic days per week and clinicians seeing a higher number of new patients per week.

We have added this additional analysis in our manuscript as below.

Methods lines 206-214: ***In a secondary analysis, we used logistic regression to measure the impact of various clinician-level variables on the likelihood of a patient receiving an SIC in both the pre-intervention and intervention periods. The logistic regression was conducted at the level of the patient-wedge with the outcome of SIC receipt. Patient covariates included in the model were patient sex, age, race, insurance status, marital status, and Charlson Comorbidity Index. Clinician-level variables included in model were the number of days in clinic per week, percentage of new patients per week, average patients per week, average encounters per day, years in practice, and end-of-life quality metrics (hospice enrollment rate, inpatient death rate, and chemo utilization at the end of life). All analyses were conducted using R version 3.6.0.***

Results lines 423-425: ***In our secondary regression analysis, specialist oncologists, higher number of days per week in clinic, and higher percentage of new patients per week were associated with significantly greater likelihood of conducting an SIC with a patient (S3 Table).***

***S3 Table: logistic regression at the patient-wedge level identifying clinician characteristics associated with increased likelihood of conducting an SIC:***

|  | OR | (95% CI) | | P |
| --- | --- | --- | --- | --- |
| **Sex** |  |  |  |  |
| *Female (ref)* | --- | --- | --- | --- |
| *Male* | 0.94 | 0.80 | 1.11 | 0.48 |
| **Age** |  |  |  |  |
| *18-35 (ref)* | --- | --- | --- | --- |
| *36-50* | 2.11 | 1.23 | 3.87 | 0.01 |
| *51-64* | 2.33 | 1.40 | 4.18 | 0.00 |
| *65+* | 2.18 | 1.27 | 4.00 | 0.01 |
| **Race** |  |  |  |  |
| *Black* | --- | --- | --- | --- |
| *White* | 1.05 | 0.85 | 1.29 | 0.67 |
| *Other* | 1.35 | 0.77 | 1.40 | 0.77 |
| **Insurance** |  |  |  |  |
| *Commercial* | --- | --- | --- | --- |
| *Medicaid* | 1.17 | 0.84 | 1.60 | 0.34 |
| *Medicare* | 1.29 | 1.03 | 1.63 | 0.03 |
| **Marital status** |  |  |  |  |
| *Married* | --- | --- | --- | --- |
| *Single* | 1.29 | 1.04 | 1.59 | 0.02 |
| *Other* | 1.35 | 1.11 | 1.64 | 0.00 |
| **CCI** |  |  |  |  |
| *0.00* | --- | --- | --- | --- |
| *1.00* | 1.99 | 0.55 | 7.20 | 0.28 |
| *>1* | 7.21 | 3.28 | 20.37 | 0.00 |
| **Oncology team** |  |  |  |  |
| *General (ref)* | --- | --- | --- | --- |
| *Breast* | 0.65 | 0.38 | 1.10 | 0.11 |
| *GI* | 3.40 | 2.29 | 5.04 | 0.00 |
| *GU* | 0.95 | 0.50 | 1.77 | 0.88 |
| *Lymphoma* | 1.81 | 1.10 | 2.92 | 0.02 |
| *Melanoma* | 3.73 | 1.93 | 7.20 | 0.00 |
| *Myeloma* | 2.11 | 1.13 | 3.92 | 0.02 |
| *Neuro* | 1.70 | 0.90 | 3.15 | 0.10 |
| *Thoracic* | 2.92 | 1.83 | 4.66 | 0.00 |
| **Clinician number of days in clinic per week** | 1.35 | 1.12 | 1.62 | 0.00 |
| **Clinician percentage of new patients per week** | 34.23 | 5.19 | 227.55 | 0.00 |
| **Clinician average patients per week** | 1.01 | 0.99 | 1.03 | 0.57 |
| **Clinician average encounters per day** | 0.98 | 0.91 | 1.06 | 0.68 |
| **Clinician years in practice** | 0.98 | 0.97 | 0.99 | 0.00 |
| **Clinician hospice enrollment rate** | 1.02 | 1.01 | 1.03 | 0.00 |
| **Clinician inpatient death rate** | 1.01 | 0.99 | 1.03 | 0.22 |
| **Clinician chemo rate** | 1.00 | 0.99 | 1.02 | 0.67 |

Sincerely,

Ravi B. Parikh and Eric H. Li
